# *Ex Vivo* Confocal Spectroscopy of Autofluorescence in Age-Related Macular Degeneration

**DOI:** 10.1371/journal.pone.0162869

**Published:** 2016-09-15

**Authors:** Joel Kaluzny, Patryk Purta, Zach Poskin, Jeremy D. Rogers, Amani A. Fawzi

**Affiliations:** 1 Department of Ophthalmology, Feinberg School of Medicine, Northwestern University, Chicago, Illinois, United States of America; 2 Department of Biomedical Engineering, University of Wisconsin-Madison, Madison, Wisconsin, United States of America; Medical University of South Carolina, UNITED STATES

## Abstract

**Purpose:**

We investigated the autofluorescence (AF) signature of the microscopic features of retina with age-related macular degeneration (AMD) using 488 nm excitation.

**Methods:**

The globes of four donors with AMD and four age-matched controls were embedded in paraffin and sectioned through the macula. Sections were excited using a 488 nm argon laser, and the AF emission was captured using a laser scanning confocal microscope (496–610 nm, 6 nm resolution). The data cubes were then analyzed to compare peak emission spectra between the AMD and the controls. Microscopic features, including individual lipofuscin and melanolipofuscin granules, Bruch’s Membrane, as well macroscopic features, were considered.

**Results:**

Overall, the AMD eyes showed a trend of blue-shifted emission peaks compared with the controls. These differences were statistically significant when considering the emission of the combined RPE/Bruch’s Membrane across all the tissue cross-sections (p = 0.02).

**Conclusions:**

The AF signatures of *ex vivo* AMD RPE/BrM show blue-shifted emission spectra (488 nm excitation) compared with the control tissue. The magnitude of these differences is small (~4 nm) and highlights the potential challenges of detecting these subtle spectral differences *in vivo*.

## Introduction

Age-related macular degeneration (AMD) is a leading cause of blindness. [1, 2] Late stages of this disease are characterized by irreversible visual compromise due to the loss of photoreceptors. [3] Photoreceptor loss is associated with changes in the complex system supporting their function, although cause and effect remain a subject of great dispute. [4–6] This support system includes the retinal pigment epithelium (RPE), a monolayer of pigmented cells that exchange metabolites with the neighboring photoreceptor cells, and recycle chromophores critical to the visual cycle. [7] To perform these functions, the RPE is supported by Bruch’s Membrane (BrM) and the highly vascular choriocapillaris, together providing the RPE with structural support as well as the exchange of nutrients and oxygen. Pathologic changes in the RPE/BrM complex are seen in the early stages of AMD and include thickening of BrM, [3] and the deposition of lipoproteinaceous extracellular debris, known as drusen, between the RPE and BrM. [8]

The ability to follow certain aspects of these pathological changes *in vivo* has been enhanced by the development of fundus autofluorescence (FAF) imaging. In 1995, Delori et al. introduced FAF to study lipofuscin *in vivo*, [9] while von Ruckmann et al. demonstrated that scanning laser ophthalmoscopy (SLO) could be used to study FAF. [10, 11] Since then, numerous studies have used FAF imaging modalities to study the retina and its components both *in vivo* and *ex vivo*. [4, 12–14]

Our goal is to study *ex vivo* the AF signatures of BrM and RPE and their relative contribution to the overall FAF signature in eyes with dry AMD compared with control eyes. This work is an extension of previous work documenting the curious finding of a 15 nm autofluorescence emission difference in RPE cells between AMD and control tissue at 364 nm excitation, but not at 488 nm excitation. [12] Unfortunately, the ocular transmission of both native lenses and modern ocular implants make 364 nm excitation not clinically useful, and thus we sought to revisit this question using higher spectral and spatial resolution approaches at 488 nm excitation; this would be a first step towards developing clinical tools for FAF spectroscopy *in vivo*. Here, we take advantage of higher spectral resolution combined with least-squares regression analysis to improve the spectral sensitivity, and then, using a higher spatial resolution, we examine the spectral contribution of the different component fluorophores within the RPE to the overall RPE/BrM AF signature in AMD and control tissue.

## Methods

This research was considered by the Institutional Review Board of Northwestern University and granted an exemption.

### Donor Eye Demographics

**Fig 1:**
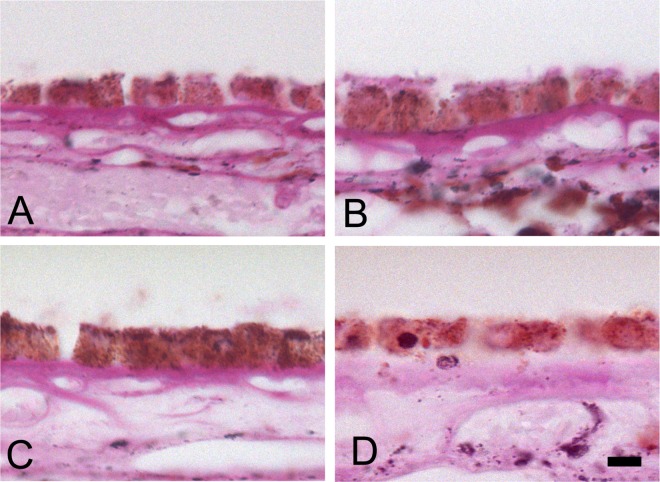
Control tissue histology. Healthy RPE cells display cuboidal morphology atop Bruch’s Membrane. A-D from the macula of each of four control donors (periodic acid Schiff: A-D, x40). Scale bar in D is 10 microns and valid for all frames.

**Fig 2:**
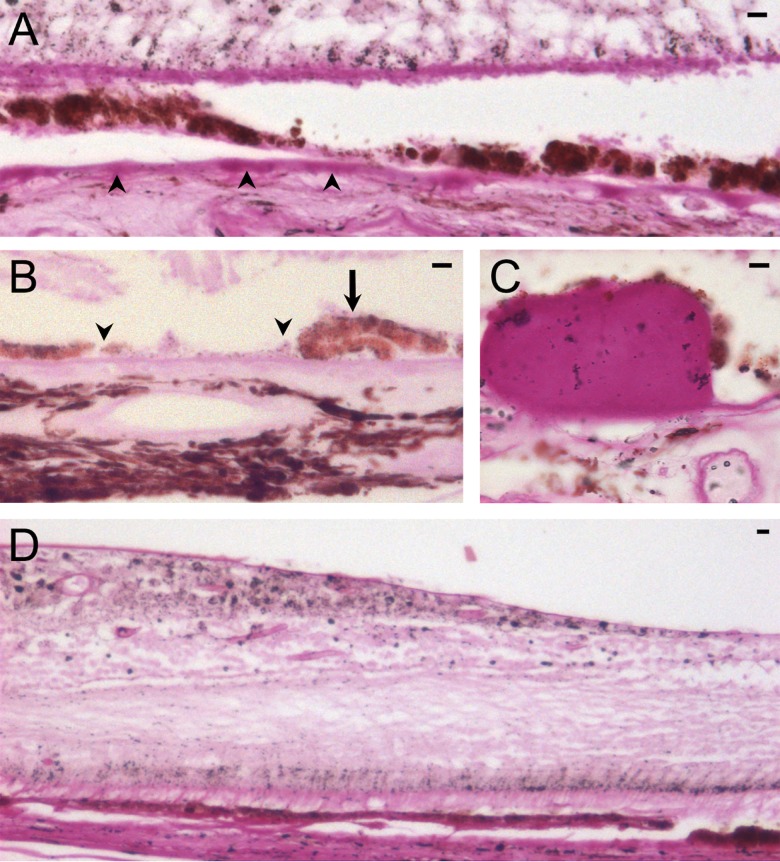
AMD classification was based on documented clinical history and confirmed on histology. Microscopic changes observed in the macula include: diffuse sub-RPE deposits (arrowheads, A), RPE atrophy (A, B), RPE clumping (arrow, B), RPE loss (between arrowheads, B), large drusen (C), and perifoveal RPE attenuation (D) (periodic acid Schiff: A & B, x20; C, x40; D, x10). Scale bar in each panel is 10 microns.

Donor eyes were obtained from the Illinois/Midwest Eye Banks. Histologic examination of the control eyes was performed to rule out subclinical AMD pathology (Fig 1). The diagnosis of dry AMD was based on the following criteria: (1) Review of clinical history and clinical ophthalmology charts; (2) Pathologic evidence of AMD changes within the macula, confirmed on gross microscopy and PAS staining (Fig 2); and (3) Lack of evidence of active choroidal neovascularization, subretinal fibrosis, or clinical history of treatment for neovascular AMD. As shown in Table 1, the average age of the AMD donors was 73.0 years, while the average age of the control donors was 72.8 years; all donors were Caucasian. The average time to fixation was 10.4 hours (max = 16.5 hours).

**Table 1:** Donor eye information.

Specimen	Donor Age	Sex, Race	Cause of Death	AMD	Time to Fix (hrs)	# of Slides	Images @ 488 nm	
							Macula	Periphery
AMD-3	67	M Caucasian	Lung Cancer	Dry AMD	6.8	5	13	8
AMD-4	66	M Caucasian	Respiratory Failure	Dry AMD	16.5	4	12	7
AMD-5	68	M Caucasian	Myocardial Infarction	Dry AMD	14	3	8	5
AMD-6	91	F Caucasian	Intracranial Hemorrhage	Dry AMD	4.3	2	9	8
C-1	71	M Caucasian	Myocardial Infarction	No	9.3	3	14	8
C-2	72	F Caucasian	Brain Cancer	No	8	2	8	7
C-3	74	F Caucasian	COPD	No	8	2	8	8
C-6	75	F Caucasian	Ovarian Cancer	No	9	2	6	6

Abbreviations: AMD (age-related macular degeneration), COPD (chronic obstructive pulmonary disease)

### Specimen Preparation and Histopathology

Globes were fixed in 10% neutral buffered formalin. Eyes were oriented based on the location of the superior and inferior oblique muscles. Horizontal cuts removed the superior and inferior calottes 10 mm on either side of the optic nerve, resulting in a center calotte containing the optic nerve, macula, and pupil. Central calottes were embedded in paraffin wax. Seven μm-thick histological cross-sections of the macular region were placed on slides. The peripheral retinal regions were analyzed from the periphery of the same sections that traversed the macula (i.e., temporal and nasal retinal periphery).

Sections were de-paraffinized in two cycles of xylene for 20 minutes each and subsequently rehydrated in two cycles of 100% ethanol for 10 minutes each, followed by one cycle of 95% ethanol and 70% ethanol for two minutes each. For autofluorescence analysis, the slides were embedded with Prolong Gold Antifade Reagent (Life Technologies, Grand Island, NY, USA) and covered with cover slips. For histopathologic evaluation of the tissue, we stained additional cross-sections with Periodic Acid-Schiff (PAS). These sections (Figs 1 and 2) were selected within 25 microns of the unstained slides used for autofluorescence.

### Confocal Microscopy and Autofluorescence Spectroscopy

Spectral autofluorescence images were collected from the unstained histological cross-sections using a 100x oil immersion, plan apochromatic TIRF, 1.5-NA objective lens on a Nikon A1R laser scanning confocal microscope (Nikon Instruments, Inc., Melville, NY, USA). The laser intensity, pinhole diameter (1.3 AU), scan speed (8X), and image size (1024 x 1024 pixels) were constant for each scan. Acquisition was controlled via Nikon Elements NIS 4.0 computer software (Nikon Instruments, Inc., Melville, NY, USA).

Datasets were acquired at 488 nm excitation and emission spectra were collected between 496 and 616 nm (21 channels at 6 nm intervals). A low-angle incidence dichroic mirror (filter) at 405/488 nm provided additional filtering to separate excitation and emission light.

Sections within the macula were confirmed by the presence of a multi-layered ganglion cell layer and proximity to the foveal section. [15] “Peripheral datasets” were obtained from the periphery (nasal or temporal) of the chosen macula slide and confirmed by a single ganglion cell layer.

To ensure that the variations in emission spectra are due to actual changes in the sample and not artifacts related to spectrometer instability, a suspension of Fluoresbrite^®^ YO Carboxylate microspheres (Polysciences Inc., Warrington, PA, USA) was imaged (using the same spectral confocal microscopy approach) at the beginning and end of each imaging session.

The autofluorescence emission datasets were exported as individual 12-bit files for each spectral channel recorded by the microscope (total 21 channels).

### Data Analysis and Normalization

**Fig 3:**
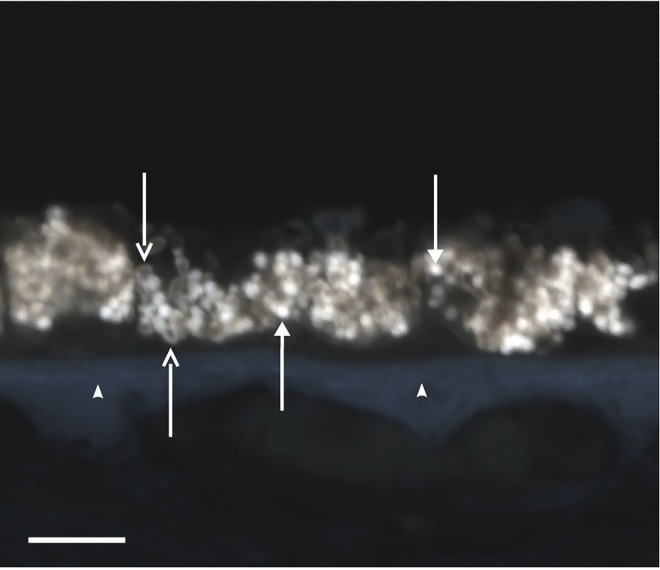
A pseudo-color representation of the spectral data cube. Arrows, melanolipofuscin granules; Solid arrows, lipofuscin granules; Arrowheads, Bruch’s Membrane. Scale bar in lower left is 10 microns.

The individual files were de-identified, imported to MATLAB (The Mathworks Inc., Natick, MA, USA) and stacked to form a spectral data cube of 1024 x 1024 pixels by 21 channels. A pseudo-color image was generated by dividing the 21 channels of the data cube into three equidistant spectral channels and averaging them to form the red, green, and blue channels of a pseudo-RGB image (Fig 3). While not spectrally accurate, this method uses the full emission spectra to visualize the qualitative spectral differences in the data cube. The pseudo-color image was then used for selecting specific components (e.g., regions of interest (ROI)) of the RPE/choroid complex for subsequent analysis.

For each ROI, a least-squares regression was used to fit a second-order polynomial to the average spectrum from 496 to 616 nm in 6 nm intervals, and the emission peak was determined analytically. A second-order polynomial was sufficient to accurately represent the spectra while minimizing the degrees of freedom in the fit. The peak wavelengths of the emission spectra were then averaged for all the pixels within an ROI.

For each imaging session, the same analysis was performed for the spectral peaks of the microspheres by averaging the peak emission obtained at the beginning and end of each session to determine a wavelength reference for correction per session. The measured microsphere emission peak was compared with the reported microsphere emission peak of 546 nm, and the emission spectra were offset accordingly to correct the instrument output for each imaging session. [16]

### Manual Microscope Spectral Analysis

**Fig 4:**
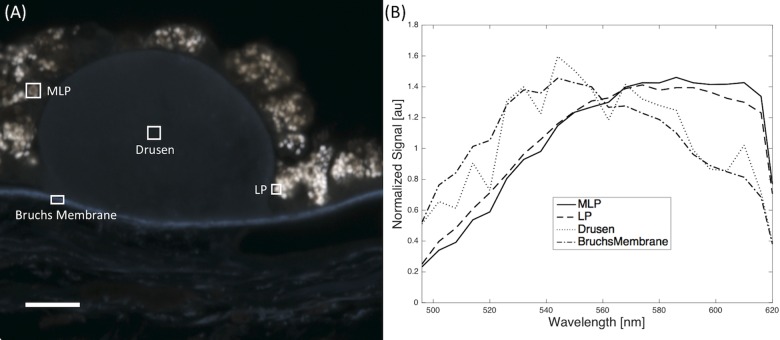
Spectral analysis of retina section. (A) An example of the ROIs selected is shown in an image with melanolipofuscin (MLP), lipofuscin (LP), Drusen, and Bruch’s Membrane. Scale bar in lower left indicates 10 microns. (B) The averaged spectra of each selection normalized by its spectral average.

Individual components of the retina were manually selected from each pseudo-color RGB image by defining the ROIs within each image. The ROIs corresponded in size with single RPE granules (either lipofuscin or melanolipofuscin, approximately 1 micron in diameter) and were selected to include the signal from each granule while minimizing the signal from ambiguous or dissimilar neighboring elements. As many as five lipofuscin and melanolipofuscin granules per cross-sectional image were selected according to their visual appearance and fluorescent intensity (Fig 3). As a guideline for these selections, we used the fluorescent ultrastructure of lipofuscin and complex melanin granules, as described by Feeney in 1978, [17] and again at higher resolution by Ach et al. in 2012. [18] The lipofuscin granules were identified by their uniform, highly fluorescent, and circular appearance. The melanolipofuscin granules appeared as hollow rings with central melanin surrounded by distinctly fluorescent lipofuscin ring. [5, 18, 19] Similarly, at least five distinct regions of BrM were selected in each image. BrM was easily identified by its strong auto-fluorescence (which appears blue in our pseudo-color representation) and its location beneath the RPE layer. Examples of spectra are shown in Fig 4, with the average spectra from each normalized to facilitate visual comparison.

The manual selection process and spectral analysis was conducted for both AMD and the control eyes; the result was a large spectral dataset of retinal components. Between two to five slides were analyzed for each eye, with multiple images taken from each slide (Table 1).

### Automated Macroscopic Spectral Analysis

In addition to comparing manually selected ROIs that sampled micro-structures of the RPE/BrM, we performed an unsupervised segmentation of the macroscopic components of the RPE/BrM for the entire cross-section. Analyzing the data in this fashion reduces noise and selection bias, and is more similar to lower-resolution, clinically relevant imaging AF methods. Since histopathologic cross-sections have variable structural components, simple averaging will mostly include dark pixels, and is therefore inappropriate. To limit the analysis to the RPE and BrM, an intensity threshold was applied to each cross-section, effectively removing all low-intensity pixels related to background noise or weak autofluorescent signal from the choroid and sclera.

**Fig 5:**
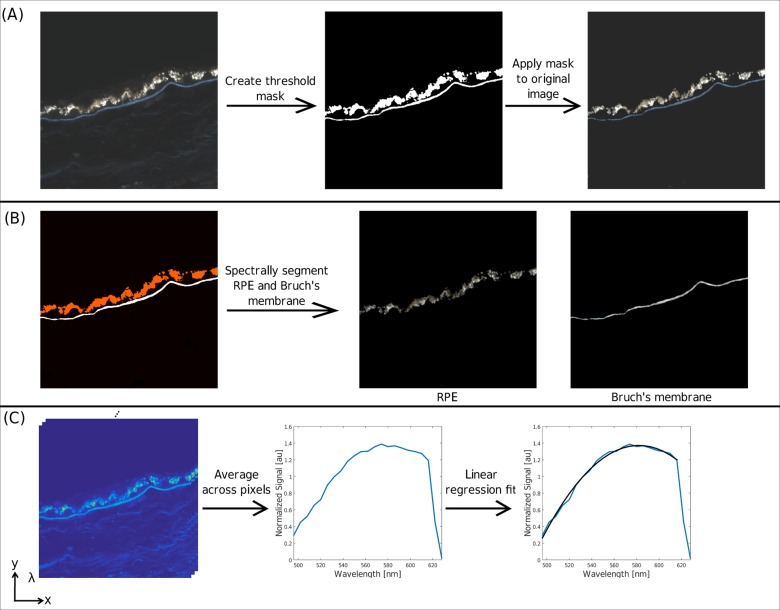
Representation of automated spectral analysis. (A) Thresholding of original image to remove low-intensity pixels and weak autofluorescent signal from the choroid and sclera. (B) Result of image segmentation to isolate RPE or Bruch’s Membrane. (C) The emission peak is determined by averaging the spectra of all pixels of interest and applying a linear regression fit.

Further image segmentation was performed to separate the RPE from BrM based on differences in their spectral emission peak. To achieve this, we exploited the spectral emission differences between the BrM and RPE, namely, that pixels within the BrM emission are more intense within the green spectral range (526 to 538 nm), whereas RPE pixels are more intense within the orange spectral range (598 to 610 nm). To quantify these differences, the pixel intensities from the orange spectral range were subtracted from the intensities within the green spectral range, resulting in a mask image in which the BrM pixels had a positive value and the RPE pixels had a negative value. The images were then reconstructed to isolate the RPE or BrM for further analysis. The relative area of BrM to RPE was determined by dividing the number of BrM pixels by the number of RPE pixels, whereas the relative intensity of BRM to RPE was determined by comparing the average pixel intensity in each component. The image processing is summarized in Fig 5.

### Statistical Analysis

The variability in the mean of the peak wavelength, relative intensity, and relative area for each individual eye was determined by the standard error of the mean (SEM). The significance of the spectral peak, relative intensity, and relative area differences between the AMD-affected eyes and the control eyes was determined using a two-tailed student’s *t*-test, assuming independent random samples with equal but unknown variances.

## Results

### Manual Microscopic Spectral Analysis

Overall, compared with the control eyes, the individual microscopic components (lipofuscin granules, melanolipofuscin granules, BrM) showed a similar trend of blue-shifted peak emission in AMD, but the differences were not statistically significant. A complete list of the results and significance tests are summarized in Table 2.

**Table 2:** Results Summary.

		AMD	Control	P-value
*Manual Microscope Spectra Analysis*			* *	* *
Peak wavelength of Lipofuscin (nm) ±SEM	Macula	570.5 ± 2.7	574.2 ± 2.7	0.1
	Periphery	572.4 ± 1.4	576.1 ± 3.9	0.13
Peak wavelength of Melanolipofuscin (nm) ±SEM	Macula	575.9 ± 2.8	578.6 ± 3.5	0.27
	Periphery	581.1 ± 2.8	583.7 ± 6.4	0.48
Peak wavelength of Bruch’s Membrane (nm) ±SEM	Macula	547.2 ± 1.3	548.2 ± 2.1	0.45
	Periphery	546.1 ± 1.3	547.7 ± 1.2	0.12
*Automated Microscope Spectral Analysis*			* *	* *
Peak wavelength of combined RPE and Bruch's Membrane (nm) ±SEM	Macula	567.6 ± 2.8	572.1 ± 2.9	0.07
	Periphery	568.5 ± 0.7	572.7 ± 2.9	0.03
	Combined	568.0 ± 1.2	572.4 ± 2.2	0.02
Peak wavelength of RPE (nm) ±SEM	Macula	572.5 ± 2.7	576.0 ± 4.0	0.21
	Periphery	575.3 ± 1.8	577.9 ± 4.2	0.3
	Combined	573.7 ± 2.0	576.9 ± 4.0	0.2
Peak wavelength of Bruch's Membrane (nm) ±SEM	Macula	548.6 ± 1.9	549.0 ± 1.2	0.75
	Periphery	546.6 ± 1.3	548.7 ± 1.4	0.07
	Combined	547.7 ± 1.3	548.8 ± 1.3	0.27
Relative area of Bruch's Membrane to RPE ±SEM	Macula	0.56 ± 0.17	0.35 ± 0.08	0.07
	Periphery	0.41 ± 0.07	0.34 ± 0.09	0.29
	Combined	0.50 ± 0.12	0.35 ± 0.05	0.07
Relative intensity of Bruch's Membrane to RPE ±SEM	Macula	0.37 ± 0.05	0.33 ± 0.06	0.35
	Periphery	0.56 ± 0.02	0.42 ± 0.11	0.06
	Combined	0.45 ± 0.03	0.38 ± 0.07	0.12

Abbreviations: ROI (region of interest), SEM (standard error of the mean), RPE (retina pigment epithelium)

### Automated Microscopic Spectral Analysis

**Fig 6:**
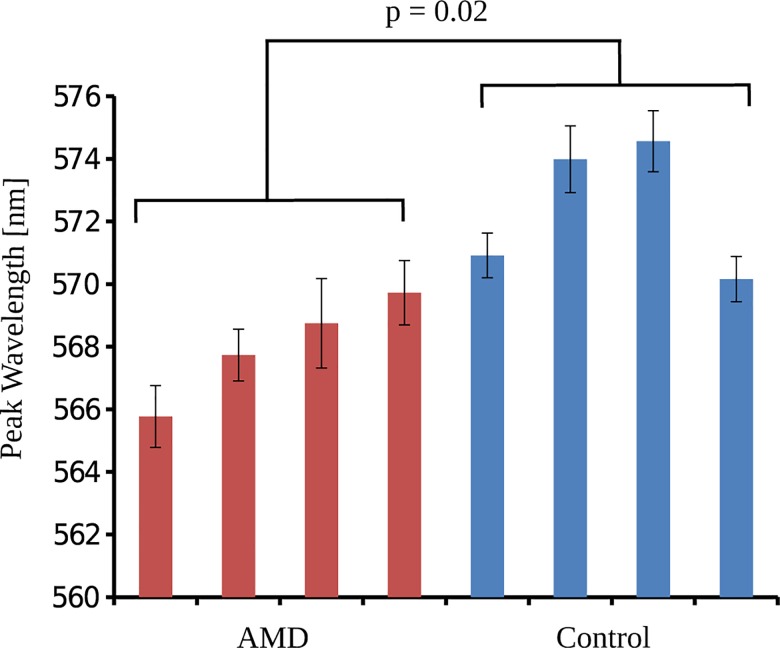
The AF emission peak wavelength for each eye. The bars indicate the combined RPE and BrM AF emission peak wavelength of each eye (red = AMD, blue = control). The average AF peak wavelength of the AMD group was significantly lower compared with controls, with *p* = 0.02. Abbreviations: AF (autofluorescence).

We first considered the overall spectral autofluorescence of the combined RPE and BrM, and the peak emission wavelength was determined for each individual eye (Table 2). The average emission peak for AMD eyes (overall macula and periphery) was 568.0 ± 1.2 nm compared with 572.4 ± 2.2 nm for the control eyes (*p* = 0.02, two-tailed *t*-test; Fig 6). We then compared the peak wavelength analysis for the macula and periphery, separately, and found a significant blue shift in the periphery (*p* = 0.03) and a trend in the macula (*p* = 0.07) in AMD eyes.

For the automatically segmented combined RPE and BrM (Fig 5), we found that the emission peak wavelength for AMD eyes was generally lower than that of the control eyes (p = 0.02). For either RPE or BrM separately, these differences were not significant (*p* = 0.20 for RPE; *p* = 0.27 for BrM). Similarly, a comparison of the RPE and BrM emission peak wavelengths between the macula and periphery revealed no significant differences.

Next, we compared the relative area and the relative intensity of the BrM to the RPE in each cross-section. The average relative area of BrM to RPE was 0.50 ± 0.12 for the AMD eyes and 0.35 ± 0.05 for the control eyes (*p* = 0.07). In general, the relative fluorescence intensity of BrM compared with RPE was greater in the AMD eyes compared with the control eyes, but the difference was not significant (*p* = 0.12). We further compared the relative intensities and areas of BrM and RPE within the macula or periphery between groups, but no significant differences were observed.

## Discussion

Our results show a statistically significant spectral difference between the AMD RPE/BrM compared with the controls when considering the combined RPE+BrM autofluorescence at 488 nm excitation (Table 2, Fig 6). While the difference is subtle, the spectral signature at this wavelength is sensitive to many cellular changes that are relevant to the proposed mechanisms of AMD. Actively studied AF emitters include bis-retinoid fluorophores (e.g., A2E), extra-cellular matrix components, and multiple unidentified constituents, studied in the retina and in other human tissue, that contribute to the overall signal. [20–22] The observations made here are in line with autofluorescence changes expected in AMD pathology.

### Pathologic Significance of AF Spectral Differences

RPE autofluorescence increases with age [10, 13, 23, 24] and it’s absence on FAF imaging has been used as a marker of RPE atrophy in AMD. The main RPE fluorophore is the lipofuscin granules, [25] and these intracellular aggregates and their bisretinoid components (e.g. A2E) have been traditionally thought to contribute to RPE cell dysfunction by generating phototoxic reactive oxygen species, aldehyde reactive species, and advanced glycosylation end product adducts of cellular structures. [22, 26–29] Similarly, melanolipofuscin is another RPE fluorophore [19] that is thought to be more abundant in AMD. [23]

Previous studies have shown a blue shift (of approximately 30 nm) in the autofluorescence emission of RPE cell extracts of AMD compared with control eyes; [30] this shift is thought to reflect changes in the fluorescent properties of oxidized vs. non-oxidized fluorophores. The difference in magnitude between our findings and previous studies might be explained by the differences in the excitation wavelength used (430 nm vs. 488 nm) and/or tissue processing (whole tissue vs. RPE extract). This disparity could also mean that while there is a strong difference in chemically purified RPE extracts, only a minor trend exists at the RPE granule level. [12]

There are several pathologic changes in BrM that could result in a blue shift in spectral emission. During normal aging, BrM undergoes thickening due to increased deposition of lipids, glycosaminoglycans, elastin, and collagen [31–34], which may play a role in the eventual dysfunction seen in AMD. [35, 36] In AMD, additional lipid accumulation results in characteristic basal linear deposits (BlinD) and large drusen [8]; and their accumulation contributes to disruption of the transfer of nutrients across BrM. [37] Decreased permeability of BrM may also result from an imbalance in metalloproteinases and their tissue inhibitors, leading to the disruption of the normal turnover of the extracellular matrix within BrM [38–41]. While the autofluorescence signatures of collagen and elastin are usually examined using UV excitation, studies from bronchial tissue using 488 nm excitation have shown that elastin has a peak emission wavelength of 530 nm [21, 42] and that type 1 collagen emits less intensely at 560 nm.

Additionally, the relative area of BrM and RPE will have an effect on the combined spectra of the two. From our component-level analysis, we found that BrM had a ~26 nm shorter peak emission wavelength than RPE granules (549 nm vs. 575 nm); therefore, an increase in the amount of BrM relative to the RPE would result in a relative blue shift of the combined BrM+RPE emission. We examined these effects by looking at the area and intensity of BrM emission relative to the RPE and observed a trend towards an increased contribution of BrM in both area and intensity for AMD, as shown in Table 2.

### Individual Component Analysis

We found no statistically significant differences in peak emission spectra between the AMD and control when considering the RPE cell layer, BrM, or the individual granules (lipofuscin and melanolipofuscin) in isolation. This was true for manually analyzed components, as well as with our automated segmentation strategy. Despite the lack of significance, the spectral trends in each of these structures were consistent with the combined RPE+BrM macroscopic results. It is conceivable that the statistically significant results seen when considering RPE+BrM are the result of three complementary trends: a blue shift in RPE granules, a blue shift in BrM, and a blue shift caused by an increased area of BrM relative to RPE. Perhaps with a larger sample size, these individual contributions would become more significant. These results are relevant as they indicate that there is not one single component that is driving the overall shift, but likely a more modest, diffuse change happening across the RPE+BrM.

### Clinical Significance

While statistically significant, the spectral differences we have observed are very subtle and emphasize the precision required to differentiate AMD from the control eyes based on AF emission spectra alone. Our results are lower in magnitude, but follow the same trend noted by Marmorstein in the RPE cells of AMD tissue using 364 nm excitation (15 nm blue shift). [12] In contrast to our findings, these authors were not able to detect spectral differences at 488 nm excitation, which may have been related to the limited spectral resolution (10 nm) of their approach. Since UV excitation at 364 nm cannot be used clinically due to safety issues and limited ocular penetration, differences that are detectable at 488 nm would have important clinical implications. By using 488 nm excitation and a more sensitive technique (e.g., higher-resolution spectrometry with least-squares regression analysis), our experiment was able to show a spectral difference in dry AMD at a clinically relevant wavelength. However, utilizing this technique for *in vivo* measurements will be complicated by other sources of AF signal in the eye. Notably, Delori has previously described a 520–580 nm peak emission, which was hypothesized to orginate from the neurosensory retina. [9] Further contributions from the lens and retina may obscure the subtle changes we have reported. While newer AF techniques have shown the ability to differentiate retina features *in vivo* in AMD, there has not been any report of these techniques being used to differentiate microscopic retina components in AMD from control. [43, 44]

### Strengths and Weaknesses

The strengths of our study include the improved sensitivity in both the spatial and spectral domains compared with previous similar work.

Because the spectral detector collects the emission spectra at 6 nm intervals, the resolution of the detector limits the ability to discern minute changes in the emission peak between samples. By fitting the emission spectra through least-squares regression, an estimate of the peak can be determined with greater precision than the spectral detector. Furthermore, by averaging the peak wavelength for a collection of spectra in a given sample, we were able to improve the accuracy of the least-squares regression fit, effectively improving the accuracy of our interpolate between data points. Our intricate manual selection of retinal components (i.e., RPE granules and BrM) also enabled us to compare these elements separately before analyzing their combined effects.

Comparison of these results with previous reports of the peak AF emission wavelength of lipofuscin in healthy controls reveals a lack of agreement in the literature, with reported peaks ranging from 555 nm to greater than 600 nm. [9, 12, 45] These discrepancies highlight the sensitivity of this absolute measurement to experimental factors such as excitation wavelength, specimen preparation, and imaging environment (*in vivo* vs. *ex vivo*). While the accuracy of the absolute peak was less critical for our relative change analysis, it does limit the our ability to compare results with other experiments.

The limited sample size and statistical power likely affected our comparisons. Perhaps with a larger sample, the trends we observed (e.g., blue shift of RPE granules) would prove to be significant. Additionally, translating these detection techniques to an *in vivo* process will require solving several additional issues related to *in vivo* detection (e.g., added background noise, lens autofluorescence, exposure limits) that are beyond the scope of this *ex vivo* study.

## Conclusion

It is very exciting that the spectral differences we observed, while subtle, are tightly related to the complex pathologic changes in AMD. It is conceivable that FAF spectral analysis could still be translated to clinical applications using higher-sensitivity spectral detectors. More elaborate detection and analytical techniques may be needed to detect these differences *in vivo*, and could ultimately lead to new diagnostic tools that can predict progression to AMD in aging eyes.

## References

[1] Klein R, Klein BE, Linton KL. Prevalence of age-related maculopathy. The Beaver Dam Eye Study. Ophthalmology. 1992;99(6):933–43. . 1630784 10.1016/s0161-6420(92)31871-8

[2] Jonasson F, Arnarsson A, Eiriksdottir G, Harris TB, Launer LJ, Meuer SM, et al Prevalence of age-related macular degeneration in old persons: Age, Gene/environment Susceptibility Reykjavik Study. Ophthalmology. 2011;118(5):825–30. 10.1016/j.ophtha.2010.08.044 21126770 PMC3087833

[3] Green WR, Enger C. Age-related macular degeneration histopathologic studies. The 1992 Lorenz E. Zimmerman Lecture. Ophthalmology. 1993;100(10):1519–35. . 7692366 10.1016/s0161-6420(93)31466-1

[4] Sparrow JR, Boulton M. RPE lipofuscin and its role in retinal pathobiology. Exp Eye Res. 2005;80(5):595–606. 10.1016/j.exer.2005.01.007 . 15862166

[5] Warburton S, Davis WE, Southwick K, Xin H, Woolley AT, Burton GF, et al Proteomic and phototoxic characterization of melanolipofuscin: correlation to disease and model for its origin. Mol Vis. 2007;13:318–29. 17392682 PMC2642915

[6] Curcio CA, Johnson M, Huang JD, Rudolf M. Apolipoprotein B-containing lipoproteins in retinal aging and age-related macular degeneration. J Lipid Res. 2010;51(3):451–67. 10.1194/jlr.R002238 19797256 PMC2817575

[7] Strauss O. The Retinal Pigment Epithelium in Visual Function. Physiol Rev. 2005;85(3):845–81. 15987797 10.1152/physrev.00021.2004

[8] Curcio CA, Millican CL. Basal linear deposit and large drusen are specific for early age-related maculopathy. Arch Ophthalmol. 1999;117(3):329–39. . 10088810 10.1001/archopht.117.3.329

[9] Delori FC, Dorey CK, Staurenghi G, Arend O, Goger DG, Weiter JJ. In vivo fluorescence of the ocular fundus exhibits retinal pigment epithelium lipofuscin characteristics. Invest Ophthalmol Vis Sci. 1995;36(3):718–29. . 7890502

[10] von Ruckmann A, Fitzke FW, Bird AC. Fundus autofluorescence in age-related macular disease imaged with a laser scanning ophthalmoscope. Invest Ophthalmol Vis Sci. 1997;38(2):478–86. Epub 1997/02/01. . 9040481

[11] von Ruckmann A, Fitzke FW, Bird AC. Distribution of fundus autofluorescence with a scanning laser ophthalmoscope. Br J Ophthalmol. 1995;79(5):407–12. Epub 1995/05/01. ; PubMed Central PMCID: PMCPmc505125. 7612549 10.1136/bjo.79.5.407 PMC505125

[12] Marmorstein AD, Marmorstein LY, Sakaguchi H, Hollyfield JG. Spectral profiling of autofluorescence associated with lipofuscin, Bruch's Membrane, and sub-RPE deposits in normal and AMD eyes. Invest Ophthalmol Vis Sci. 2002;43(7):2435–41. . 12091448

[13] Greenberg JP, Duncker T, Woods RL, Smith RT, Sparrow JR, Delori FC. Quantitative fundus autofluorescence in healthy eyes. Invest Ophthalmol Vis Sci. 2013;54(8):5684–93. 10.1167/iovs.13-12445 23860757 PMC3759220

[14] Burke TR, Duncker T, Woods RL, Greenberg JP, Zernant J, Tsang SH, et al Quantitative fundus autofluorescence in recessive Stargardt disease. Invest Ophthalmol Vis Sci. 2014;55(5):2841–52. 10.1167/iovs.13-13624 24677105 PMC4008047

[15] Cummings TJ. Ophthalmic pathology: a concise guide New York: Springer; 2013 vii, 192 pages p.

[16] Sisken JE. Chapter 4 Fluorescent Standards In: Taylor DL, Yu-Li W, editors. Methods Cell Biol. Volume 30: Academic Press; 1989 p. 113–26. 2648108

[17] Feeney L. Lipofuscin and melanin of human retinal pigment epithelium. Fluorescence, enzyme cytochemical, and ultrastructural studies. Invest Ophthalmol Vis Sci. 1978;17(7):583–600. Epub 1978/07/01. . 669890

[18] Ach T, Best G, Rossberger S, Heintzmann R, Cremer C, Dithmar S. Autofluorescence imaging of human RPE cell granules using structured illumination microscopy. Br J Ophthalmol. 2012;96(8):1141–4. 10.1136/bjophthalmol-2012-301547 . 22760487

[19] Biesemeier A, Schraermeyer U, Eibl O. Chemical composition of melanosomes, lipofuscin and melanolipofuscin granules of human RPE tissues. Exp Eye Res. 2011;93(1):29–39. 10.1016/j.exer.2011.04.004 . 21524648

[20] Rocha R, Villaverde AB, Silveira L Jr, Brugnera A Jr, Alves LP, Munin E, et al Fluorescence and reflectance spectroscopy for identification of atherosclerosis in human carotid arteries using principal components analysis. Photomed Laser Surg. 2008;26(4):329–35. 10.1089/pho.2007.2208 . 18665764

[21] Thiberville L, Moreno-Swirc S, Vercauteren T, Peltier E, Cave C, Bourg Heckly G. In vivo imaging of the bronchial wall microstructure using fibered confocal fluorescence microscopy. Am J Respir Crit Care Med. 2007;175(1):22–31. 10.1164/rccm.200605-684OC . 17023733

[22] Sparrow JR, Gregory-Roberts E, Yamamoto K, Blonska A, Ghosh SK, Ueda K, et al The bisretinoids of retinal pigment epithelium. Prog Retin Eye Res. 2012;31(2):121–35. Epub 2012/01/03. 10.1016/j.preteyeres.2011.12.001 22209824 PMC3288746

[23] Feeney-Burns L, Hilderbrand ES, Eldridge S. Aging human RPE: morphometric analysis of macular, equatorial, and peripheral cells. Invest Ophthalmol Vis Sci. 1984;25(2):195–200. . 6698741

[24] Kim SR, Jang YP, Sparrow JR. Photooxidation of RPE lipofuscin bisretinoids enhances fluorescence intensity. Vision Res. 2010;50(7):729–36. Epub 2009/10/06. 10.1016/j.visres.2009.09.015 19800359 PMC2840058

[25] Delori FC, Staurenghi G, Arend O, Dorey CK, Goger DG, Weiter JJ. In vivo measurement of lipofuscin in Stargardt's disease—Fundus flavimaculatus. Invest Ophthalmol Vis Sci. 1995;36(11):2327–31. . 7558729

[26] Warburton S, Southwick K, Hardman RM, Secrest AM, Grow RK, Xin H, et al Examining the proteins of functional retinal lipofuscin using proteomic analysis as a guide for understanding its origin. Mol Vis. 2005;11:1122–34. . 16379024

[27] Rozanowska M, Jarvis-Evans J, Korytowski W, Boulton ME, Burke JM, Sarna T. Blue light-induced reactivity of retinal age pigment. In vitro generation of oxygen-reactive species. J Biol Chem. 1995;270(32):18825–30. . 7642534 10.1074/jbc.270.32.18825

[28] Sparrow JR, Parish CA, Hashimoto M, Nakanishi K. A2E, a lipofuscin fluorophore, in human retinal pigmented epithelial cells in culture. Invest Ophthalmol Vis Sci. 1999;40(12):2988–95. . 10549662

[29] Wu Y, Yanase E, Feng X, Siegel MM, Sparrow JR. Structural characterization of bisretinoid A2E photocleavage products and implications for age-related macular degeneration. Proc Natl Acad Sci U S A. 2010;107(16):7275–80. Epub 2010/04/07. 10.1073/pnas.0913112107 20368460 PMC2867734

[30] Feldman TB, Yakovleva MA, Arbukhanova PM, Borzenok SA, Kononikhin AS, Popov IA, et al Changes in spectral properties and composition of lipofuscin fluorophores from human-retinal-pigment epithelium with age and pathology. Anal Bioanal Chem. 2014 10.1007/s00216-014-8353-z . 25471291

[31] Newsome DA, Huh W, Green WR. Bruch's membrane age-related changes vary by region. Curr Eye Res. 1987;6(10):1211–21. . 3677781 10.3109/02713688709025231

[32] Ramrattan RS, van der Schaft TL, Mooy CM, de Bruijn WC, Mulder PG, de Jong PT. Morphometric analysis of Bruch's membrane, the choriocapillaris, and the choroid in aging. Invest Ophthalmol Vis Sci. 1994;35(6):2857–64. . 8188481

[33] Okubo A, Rosa RH Jr, Bunce CV, Alexander RA, Fan JT, Bird AC, et al The relationships of age changes in retinal pigment epithelium and Bruch's membrane. Invest Ophthalmol Vis Sci. 1999;40(2):443–9. . 9950604

[34] Bhutto I, Lutty G. Understanding age-related macular degeneration (AMD): relationships between the photoreceptor/retinal pigment epithelium/Bruch's membrane/choriocapillaris complex. Mol Aspects Med. 2012;33(4):295–317. 10.1016/j.mam.2012.04.005 22542780 PMC3392421

[35] Smith W, Assink J, Klein R, Mitchell P, Klaver CC, Klein BE, et al Risk factors for age-related macular degeneration: Pooled findings from three continents. Ophthalmology. 2001;108(4):697–704. . 11297486 10.1016/s0161-6420(00)00580-7

[36] Spraul CW, Lang GE, Grossniklaus HE, Lang GK. Histologic and morphometric analysis of the choroid, Bruch's membrane, and retinal pigment epithelium in postmortem eyes with age-related macular degeneration and histologic examination of surgically excised choroidal neovascular membranes. Surv Ophthalmol. 1999;44 Suppl 1:S10–32. . 10548114 10.1016/s0039-6257(99)00086-7

[37] Mullins RF, Johnson MN, Faidley EA, Skeie JM, Huang J. Choriocapillaris vascular dropout related to density of drusen in human eyes with early age-related macular degeneration. Invest Ophthalmol Vis Sci. 2011;52(3):1606–12. 10.1167/iovs.10-6476 21398287 PMC3101687

[38] Nita M, Strzalka-Mrozik B, Grzybowski A, Mazurek U, Romaniuk W. Age-related macular degeneration and changes in the extracellular matrix. Med Sci Monit. 2014;20:1003–16. Epub 2014/06/19. 10.12659/msm.889887 ; PubMed Central PMCID: PMCPmc4072585. 24938626 PMC4072585

[39] Kamei M, Hollyfield JG. TIMP-3 in Bruch's membrane: changes during aging and in age-related macular degeneration. Invest Ophthalmol Vis Sci. 1999;40(10):2367–75. . 10476804

[40] Hussain AA, Lee Y, Zhang JJ, Marshall J. Disturbed matrix metalloproteinase activity of Bruch's membrane in age-related macular degeneration. Invest Ophthalmol Vis Sci. 2011;52(7):4459–66. 10.1167/iovs.10-6678 . 21498613

[41] Crabb JW, Miyagi M, Gu X, Shadrach K, West KA, Sakaguchi H, et al Drusen proteome analysis: an approach to the etiology of age-related macular degeneration. Proc Natl Acad Sci U S A. 2002;99(23):14682–7. 10.1073/pnas.222551899 12391305 PMC137479

[42] Bourg-Heckly G, Thiberville L, Vever-Bizet C, Viellerobe B, editors. In vivo endoscopic autofluorescence microspectro-imaging of bronchi and alveoli2008.

[43] Schweitzer D, Gaillard ER, Dillon J, Mullins RF, Russell S, Hoffmann B, et al Time-resolved autofluorescence imaging of human donor retina tissue from donors with significant extramacular drusen. Invest Ophthalmol Vis Sci. 2012;53(7):3376–86. 10.1167/iovs.11-8970 22511622 PMC3390004

[44] Schweitzer D, Quick S, Schenke S, Klemm M, Gehlert S, Hammer M, et al Comparison of parameters of time-resolved autofluorescence between healthy subjects and patients suffering from early AMD. Ophthalmologe. 2009;106(8):714–22. 10.1007/s00347-009-1975-4 . 19588156

[45] Sparrow JR, Wu Y, Nagasaki T, Yoon KD, Yamamoto K, Zhou J. Fundus autofluorescence and the bisretinoids of retina. Photochemical & photobiological sciences: Official journal of the European Photochemistry Association and the European Society for Photobiology. 2010;9(11):1480–9. Epub 2010/09/24. 10.1039/c0pp00207k 20862444 PMC4071605

